# Not afraid of low diastolic blood pressure anymore?

**DOI:** 10.18632/aging.102445

**Published:** 2019-11-07

**Authors:** Maciej Siński, Piotr Sobieraj, Jacek Lewandowski

**Affiliations:** 1 Department of Internal Medicine, Hypertension and Vascular Diseases, Medical University of Warsaw, Warsaw 02-097, Poland

**Keywords:** low diastolic blood pressure, cardiovascular risk, SPRINT trial

Epidemiologic studies have demonstrated that systolic blood pressure (SBP) is the most important factor influencing cardiovascular risk. The results of clinical studies including the Systolic Blood Pressure Intervention Trial (SPRINT) [1] preceded the changes in blood pressure goals proposed by hypertension societies. SPRINT showed that intensive lowering of the SBP to a goal of <120 mmHg could be beneficial and safe in contrast to the standard goal up to that point of <140 mmHg. In practice, it is unlikely that the SBP can be lowered without simultaneously reducing diastolic blood pressure (DBP). This may then limit how tightly the SBP can be controlled. For more than 30 years, investigators have argued as to whether lowering DBP increases the risk of myocardial infarction and coronary artery disease in patients with hypertension. From a pathophysiologic perspective, a low DBP may adversely affect myocardial perfusion because, unlike in other vascular beds, blood flow in the epicardial coronary arteries is maintained during diastole [2]. It is therefore reasonable to postulate that the positive effect on cardiovascular risk of achieving a low SBP target may be blunted by the increased hazard associated with a low DBP. Such concept was proven right in various studies however analysis of large population-based trials of individuals with high cardiovascular risk have been ambiguous.

Ongoing analysis of SPRINT findings has indicated that the increased cardiovascular risk in for participants with a low DBP may be explained by factors other than simply the DBP, such as higher age and higher prevalence of cardiovascular or chronic kidney disease. This suggests that a low DBP is not an independent risk factor for cardiovascular events [3,4]. This also seems to be the case in other studies of subjects with high cardiovascular risk [5]. In contrast to this view, however, analysis of the ONTARGET and TRANSCEND findings indicate that the benefit of intensive lowering of SBP may be limited because of concomitant DBP reduction [6]. Similar conclusions have been drawn by other investigators analyzing the SPRINT results showing that a DBP <55 mmHg recorded at a single visit was responsible for attenuation of the benefits of SBP reduction in both of the trial’s treatment arms [7]. Thus, the discussion regarding the influence of lowering the DBP continues.

**Figure 1:**
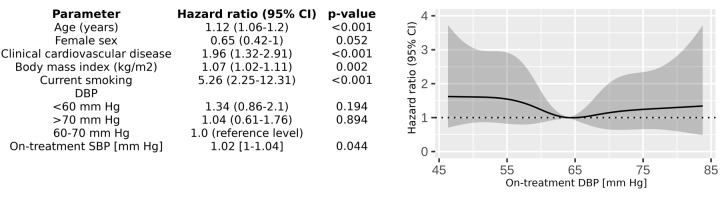
Left panel - multivariable Cox proportional hazard risk model evaluating association of on-treatment DBP with composite clinical endpoint. Right panel - hazard ratio for composite clinical endpoint according to achieved on-treatment DBP in SPRINT participants over 80 years old.

The results being highlighted here do not apply to all age groups. Only 28.2% of the patients in SPRINT were ≥75 years, and the mean age of participants in ONTARGET and TRANSCEND with a low DBP (<70 mmHg) did not exceed 70 years [6]. Elderly patients often have a high SBP but a low DBP. It is possible that cardiovascular risk in older subjects could thus be more impacted by a low DBP than in younger individuals. However, when stroke incidence in SPRINT was taken into account, an outcome measure which is highly age dependent, low DBP was not independently associated with a higher incidence of stroke [8]. We performed an additional analysis of the SPRINT study population focused on 1079 subjects >80 years old (mean age 83.4 ± 3 years). The SPRINT data were obtained from the National Heart, Lung and Blood Institute (NHLBI) Biologic Specimen and Data Repository Information Coordinating Center, but our findings are our own and do not necessarily reflect the opinions or views of the SPRINT Research Group or the NHLBI. The population we analyzed was at high risk of a clinical composite endpoint of myocardial infarction, acute coronary syndrome without infarction, stroke, acute decompensated heart failure, or cardiovascular death, of which there were 103 events (9.5%) during the trial. The risk of the composite endpoint was increased with a DBP <64 mmHg (Figure 1). However, analysis using a Cox proportional hazard risk model revealed that, after adjustment for age, sex, history of cardiovascular disease, SBP, current smoking status, and body mass index, a low DBP was not independently associated with an increased risk for the endpoint (Figure 1).

It is tempting to answer the question posed in the title positively. We have convincing evidence that a low DBP should not stand in the way of intensive blood pressure reduction. On the other hand, as clinicians, we are aware that some of our older patients faint while on hypertensive treatment, so we cannot stop asking question, “Aren’t we going too low?” Thus, there is a need for more studies specifically among elderly patients focusing on the effect of a low DBP. In a superb review concerning the J curve, Messerli and Panjrath present a figure showing that subjects with coronary artery disease who have undergone revascularization tolerate a lower DBP better than those who have not been revascularized [2]. Keeping that in mind, we should still consider low DBP as a marker of increased risk and focus on risk reduction.

DBP, diastolic blood pressure; SBP, systolic blood pressure; CI, confidence interval; composite clinical endpoint: myocardial infarction, acute coronary syndrome without infarction, stroke, acute decompensated heart failure, or cardiovascular death. DBP was included in the model at discrete variable thresholds of 60 mmHg and 70 mmHg and was not an independent risk factor.
